# A Protocol to Assess Time-of-Day-Dependent Learning and Memory in Mice Using the Novel Object Recognition Test

**DOI:** 10.21769/BioProtoc.5446

**Published:** 2025-09-20

**Authors:** Jordan Mar, Matthew A. McGregor, Vishnuvasan Raghuraman, Isabella Farhy-Tselnicker

**Affiliations:** 1 Department of Biology, Texas A&M University, College Station, TX, USA; 2 Texas A&M Institute for Neuroscience, Texas A&M University, College Station, TX, USA; 3 Department of Computer Science and Engineering, Texas A&M University, College Station, TX, USA; 4 Center for Biological Clocks Research, Texas A&M University, College Station, TX, USA

**Keywords:** Behavioral assay, Novel object recognition, Circadian rhythms/time of day, Learning and memory, Mice behavior protocol

## Abstract

Changes in learning and memory are important behavioral readouts of brain function across multiple species. In mice, a multitude of behavioral tasks exist to study learning and memory, including those influenced by extrinsic and intrinsic forces such as stress (e.g., escape from danger, hunger, or thirst) or natural curiosity and exploratory drive. The novel object recognition (NOR) test is a widely used behavioral paradigm to study memory and learning under various conditions, including age, sex, motivational state, and neural circuit dynamics. Although mice are nocturnal, many behavioral tests are performed during their inactive period (light phase, subjective night) for the convenience of the diurnal experimenters. However, learning and memory are strongly associated with the animal’s sleep-wake and circadian cycles, stressing the need to test these behaviors during the animals’ active period (dark phase, subjective day). Here, we develop a protocol to perform the NOR task during both light (subjective night) and dark (subjective day) phases in adult mice (4 months old) and provide a flexible framework to test the learning and memory components of this task at distinct times of day and associated activity periods. We also highlight methodological details critical for obtaining the expected behavioral responses.

Key features

• Enables analysis of learning and memory in mice during both active (dark) and inactive (light) phases.

• Allows for switching the time-of-day-dependent familiarization and recognition to study the impact of activity and sleep-wake cycle on cognitive performance.

• Details the environmental and experimenter-dependent conditions that can impact behavioral responses.

• Provides a flexible, adjustable platform for testing variable experimental conditions such as age, sex, learning and memory components, and genetic manipulations.

## Graphical overview


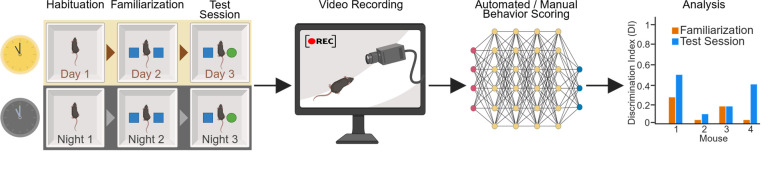


**Time-of-day-dependent novel object recognition (NOR) test.** Complete workflow of the experiment, including 3 days of testing during light or dark phases (left panel), video recording (middle panel), data analysis, and visualization of the results (right panels).

## Background

Behavioral testing in rodents enables evaluating multiple aspects of cognitive processes, including attention, learning, memory, spatial navigation, fear, and anxiety, under both normal and pathological conditions [1,2]. These tests vary in complexity and structure, with some exploiting animals’ natural instincts (such as escape from danger [3] or grooming [4]) and others employing complex training sessions to elicit learned responses (such as maze navigation [5] or pellet reaching [6]). The novel object recognition (NOR) test is routinely utilized to quantify learning and memory in mice, relying on their inherent drive to explore objects within their environment [1,7–9]. The test involves two sessions where a mouse freely explores an arena. Initially, the arena contains two identical objects for familiarization. In the second session, one object is replaced with a novel object. It is expected that the mouse's natural curiosity will lead it to spend more time exploring the novel object, providing a measure of its learning and memory. NOR is a powerful paradigm due to its high degree of flexibility and adaptation to various experimental questions, for example, to quantify short-term vs. long-term memory, or to examine how different environmental conditions (such as time of day) or genetic perturbations (such as gene knockouts) impact these cognitive processes [10,11]. Importantly, mouse behavioral performance may be influenced by stressors such as light and noise [12], underscoring the importance of carefully calibrating the testing parameters to enable reliable, measurable responses.

Cognitive function is strongly influenced by the light/dark cycle and circadian clock [13,14]. The circadian clock is an intrinsic rhythm-generating system consisting of transcriptional-translational feedback loops involving core clock genes, which maintain a 24-h periodic regulation of multiple aspects of physiology, including cognition [15–17]. Numerous studies across various species including flies, rodents, and humans have shown that learning and memory abilities vary by time of day [13,14,18–27], while circadian disruptions due to altered light/dark or feeding cycles (such as jet lag or clock gene dysregulation) result in impaired cognitive performance in several behavioral tasks [28–37], including the NOR test [10,11]. Importantly, the NOR test offers a unique advantage for studying clock-related cognitive function as it enables separate investigation of learning and memory components like consolidation and recall by varying the time of day of object familiarization and recognition. Since circadian disruptions are linked to cognitive disorders such as depression [38–40] and Alzheimer’s disease [41–43], designing robust experimental paradigms to study the underlying mechanisms is crucial for advancing our understanding of these disorders and for developing novel treatments.

Here, we describe a protocol for the NOR test that is modified [8,10,11,44] to quantify time-of-day-dependent learning and memory in mice. The test consists of three consecutive days (24 h interval) and includes habituation (day 1; no objects), familiarization (day 2; identical objects), and testing (day 3; novel object; see Graphical overview). Moreover, the habituation day serves as an “open-field test” [1] to assess time-of-day-dependent locomotion and general anxiety. This protocol is highly flexible and can be adapted to test the effects of light/dark cycle and circadian clock perturbations on learning as well as both short-term and long-term memory. This protocol also provides insights and troubleshooting suggestions to ensure robust and reproducible results.

## Materials and reagents

**Biological materials**

1. Adult (4 months old) wild-type mice (C57BL/6J background; Jax #000664) of both sexes were used for validation experiments. Sex differences in behavioral performance were not observed under these experimental conditions; thus, the data were pooled. All mice used for these experiments were born and raised in the laboratory’s mouse colony in the vivarium space.

**Reagents**

1. 200 proof pure ethanol (Koptec, catalog number: 89125-172 or similar)

**Caution:** This chemical is flammable.

**Solutions**

1. 70% ethanol

**Recipes**

**1. 70% ethanol**

**Table:** 

Reagent	Final concentration	Quantity or Volume
200 proof pure ethanol	70%	350 mL
diH_2_O	30%	150 mL

**Laboratory supplies**

1. Paper towels (Georgia-Pacific, catalog number: B0017TKE8G or similar)

2. 500 mL spray bottle (VWR, catalog number: 10216-884 or similar)

## Equipment

**Figure 1.**
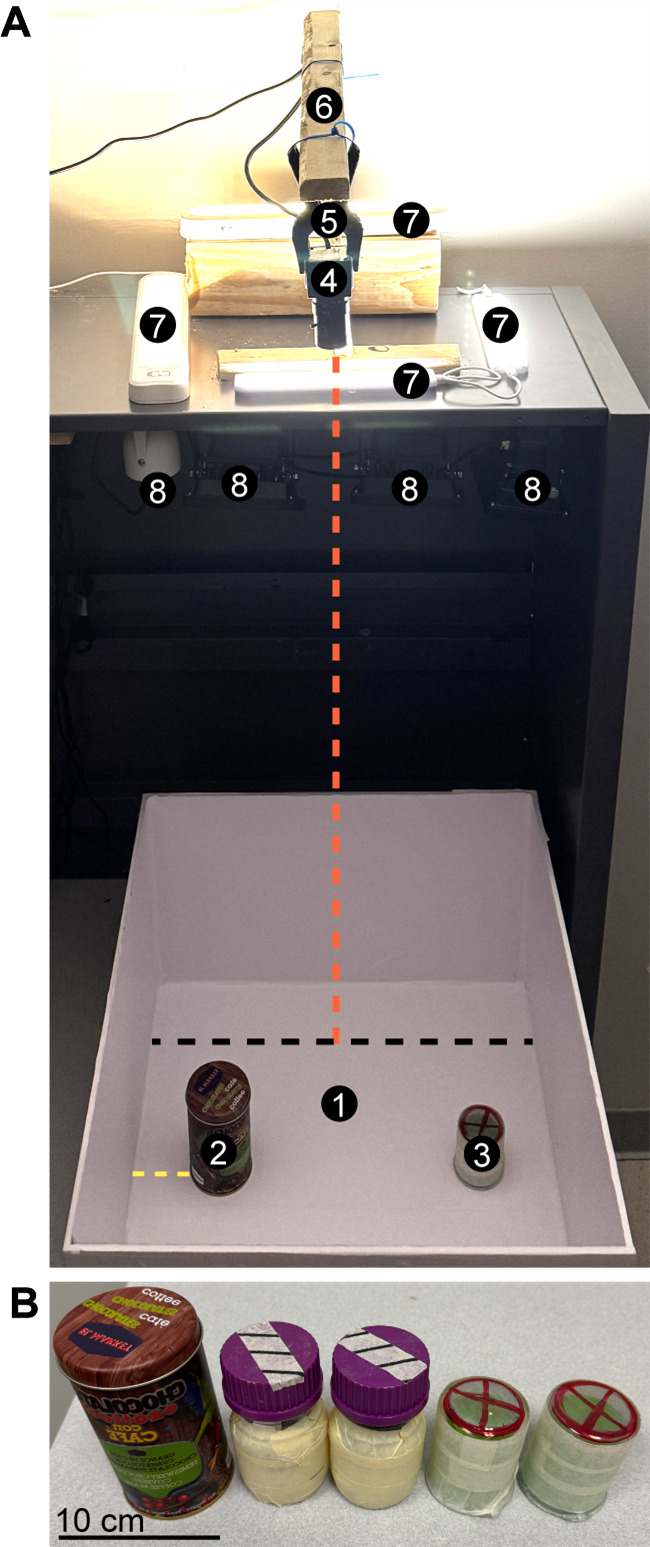
Novel object recognition (NOR) test setup. (A) Setup for behavioral testing, including the testing arena (1) shown with two different objects (2–3) placed a minimum of 5 cm away from the arena wall (yellow dashed line). The location of the objects remains constant throughout experimental days. A camera (4) is mounted above the arena (5–6: camera holders) at a distance of 96.5 cm from the arena floor (red dashed line). Camera height can be adjusted as needed, depending on camera type and setup, as long as the entire arena is within the field of view. Illuminators for both daytime (7, visible light) and nighttime (8, infrared light) are arranged as shown, avoiding direct illumination of the arena (especially important for visible light; see General notes and troubleshooting). The black dashed line corresponds to the arena width, 45 cm. (B) The different objects used for the NOR test in this protocol. On day 2 of the experiment, two identical objects are placed in the arena. One of the objects is replaced with a novel/different object on day 3 (as shown in A). Scale bar = 10 cm.

1. Testing arena size 53 L × 45 W × 30 H cm custom-made from white 7328/WRT30 cast 32% translucent acrylic sheets (Interstate Plastics, catalog number: ACRW8CPSH; see General notes)

2. Webcam (varifocal lens USB webcam, ELP, Link, or similar)

3. Wide-angle infrared (IR) illuminators (850 nm wide-angle illuminator) (Univivi, Link, or similar)

4. LED light bars (under cabinet lights) (Ezvalo, Link, or similar)

5. 4-inch spring clamp (4-inch heavy duty plastic spring clamp) (Yesker, Link, or similar)

6. Zip ties (8-inch cable ties) (Skalon, Link, or similar)

7. Overhead camera stand (custom made, see General notes; Figure 1A)

8. Objects for exploration test (see General notes and Troubleshooting section; Figure 1B): 100 mL beakers (100 mL griffin low form glass beaker, VWR, catalog number: 10754-948); 250 mL bottles (250 mL media/storage bottles with GL screw caps, VWR, catalog number: 10754-816)

9. Computer capable of recording videos (in this protocol, a Dell Optiplex desktop computer equipped with Windows 10, 32 GB RAM, 1 TB SSD, Intel Core i7 processor, and USB 3.0 ports was used)

10. Matte white wallpaper (solid white wallpaper, Homeease, Link, or similar)

11. Odor-free adhesive (removable mounting putty LJ8XP, Scotch, Link, or similar)

## Software and datasets

1. MATLAB (version R2025a, MathWorks; may be free with student license)

a. Add-on: Curve-Fitting Toolbox version 25.1

b. Add-on: Image Processing Toolbox version 25.1

c. Add-on: Signal Processing Toolbox version 25.1

d. Add-on: Statistics and Machine Learning Toolbox version 25.1

2. Python (version 3.10.16; Python Software Foundation)

3. Conda (version 24.11.3, Anaconda Software Distribution)

4. DeepLabCut (version 3.0.0 [45,46])

5. BehaviorDEPOT (version 1.6 [47])

6. Chronotate (version 1 [48])

7. Excel (Microsoft) or Google Sheets (Google, free)

8. All the code used in this protocol is available on this GitHub page

## Procedure

**Caution:** Prior to beginning experimentation, ensure that the testing arena is non-reflective (e.g., lined with matte white paper), lights are not shining directly into the arena creating bright spots, and/or IR illuminators are sufficient to produce a clear image when recorded with the video camera (see General notes and troubleshooting section for additional guidance and information).

**A. Day 1: Habituation (data collected can be used as an open-field test)**

**Figure 2.**
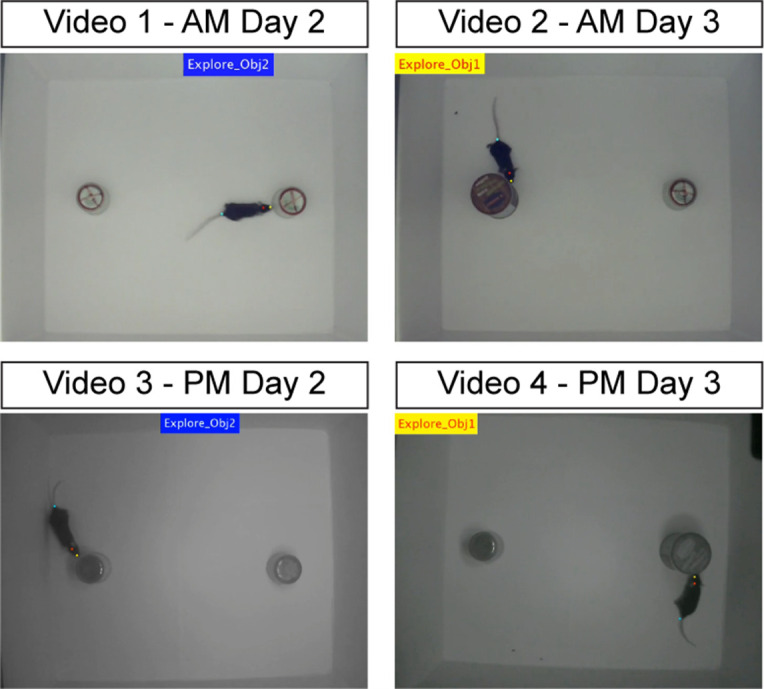
Example images of object exploration. See also Videos 1–4. Still images extracted from videos 1–4 showing a mouse within the novel object recognition (NOR) arena exploring objects on day 2 (left panels) and day 3 (right panels) during the light phase (AM; top panels) and dark phase (PM; bottom panels) as labeled. Blue and yellow banners indicate detection of an exploration bout by BehaviorDepot (see Data analysis section). During day 2 (familiarization), two identical objects are present in the arena, while during the testing session (day 3), one of the objects is replaced with a novel object (object 1, yellow banner).

**Figure 3.**
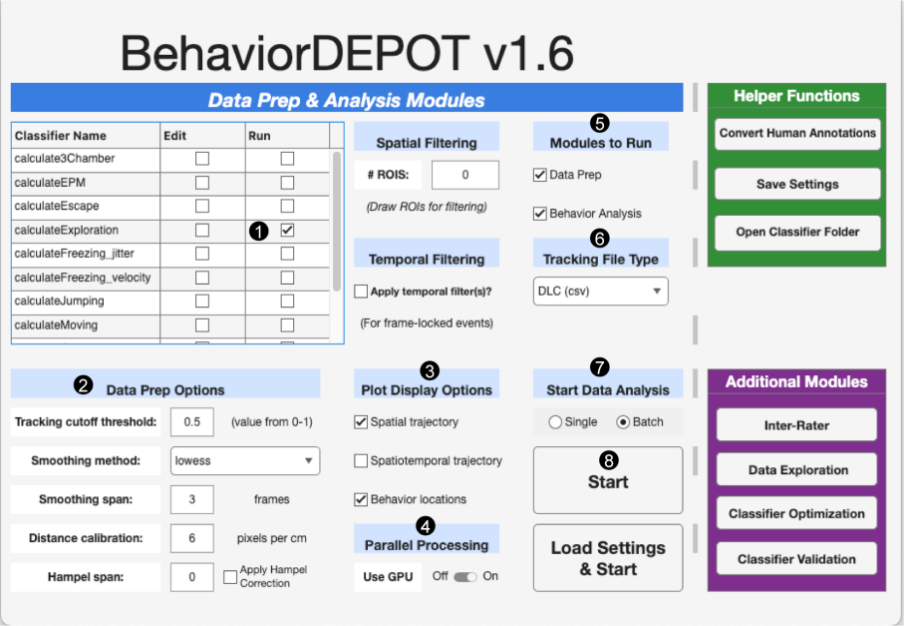
BehaviorDEPOT used for the analysis of object exploration. A screenshot of BehaviorDEPOT graphical user interface containing the steps (numbered 1–8) needed to perform exploration analysis.

**Figure 4.**
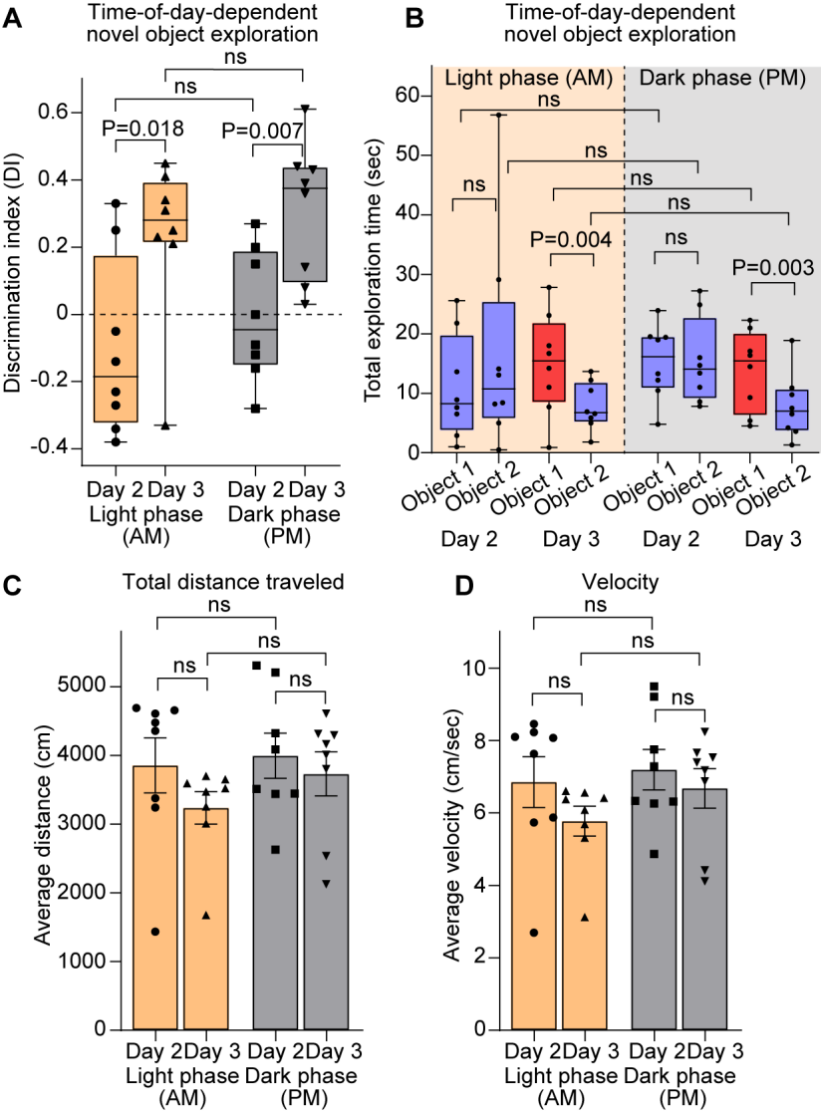
Validation of the novel object recognition (NOR) protocol. (A) Quantification of object exploration, represented as the discrimination index (DI) of mice tested during the light (yellow boxes) and dark (gray boxes) phases, as labeled. DI is higher on day 3 compared to day 2, suggesting increased exploration of the novel object over the familiar object during both activity phases. (B) Quantification of object exploration represented as total exploration time for each object (familiar objects, blue; novel object, red) in mice tested during the light (yellow background) and dark (gray background) phases. Exploration time is similar for objects 1 and 2 on day 2 (familiar objects), while exploration time is lower for the familiar object (object 2; blue box) on day 3 compared to the novel object (object 1; red box). (C–D) Kinematics parameters, including total distance traveled (C) and velocity (D), in mice tested during the light (yellow) and dark (gray) phases. No difference between the parameters is observed. N = 8 mice light phase; N = 8 mice dark phase. In A–B, data is shown as a box with whiskers (min-max), and the line is median. In C–D, data is shown as mean ± SEM. Data points represent individual mice. Statistical analysis by t-test; in B, exploration time was compared between objects within each day by a paired t-test. P value on the plots, ns = non-significant (P > 0.05). See also Figure S1.

1. Turn on the source of illumination (visible light, Figure 1A-7, or infrared light, Figure 1A-8), depending on the time of day, and ensure correct angle/brightness. Connect the camera (Figure 1A-4) to the computer and focus the lens until a clear image is obtained.

*Note: Light and camera setup must be ready* ***prior*** *to bringing the mice into the testing room.*

2. Transport mice from the vivarium to the behavioral testing room in their home cage. Obtain an additional, new (clean) cage to be used as a holding cage that will temporarily house tested mice to prevent interactions between tested and naïve mice.

3. Allow mice to acclimate to the room in their home cage for 1 h.

4. Begin video recording: Place the mouse (one at a time) into the middle of the **empty** arena facing the north wall and allow them to explore for 5 min.

*Note: This recording can be used to assess general locomotion and anxiety (as in the open-field test); however, if there is no need/requirement for this data, day 1 activity does not need to be recorded*.

5. After 5 min, stop recording and relocate the mouse from the arena to the holding cage (clean cage).

6. Wipe down the arena with 70% EtOH and paper towels and allow EtOH to air-dry (2–3 min).

7. Repeat steps A4–6 for all mice to be tested. Once complete, return mice to their home cage.

8. Return mice to the vivarium.

**B. Day 2: Familiarization (24 h post-habituation)**

1. Turn on the source of illumination (visible light, Figure 1A-7, or infrared light, Figure 1A-8), depending on the time of day, and ensure correct angle/brightness. Connect the camera (Figure 1A-4) to the computer and focus the lens until a clear image is obtained.

*Note: Light and camera setup must be ready* ***prior*** *to bringing the mice into the testing room.*

2. Transport mice from the vivarium to the behavioral testing room in their home cage. Obtain an additional, new (clean) cage to be used as a holding cage that will temporarily house tested mice to prevent interactions between tested and naïve mice.

3. Allow mice to acclimate to the room in their home cage for 1 h.

4. Place two identical objects on opposing sides of the arena (Figure 1A-2, 3); ensure ≥ 5 cm distance between the object and arena wall(s).

5. Begin video recording: Place the mouse (one at a time) into the arena (ensure the mouse is placed in the middle of the arena but facing away from the objects) and allow it to explore for 10 min.

6. After 10 min, stop recording and relocate the mouse from the arena to the holding cage (clean cage).

7. Wipe down the arena and objects with 70% EtOH and paper towel and allow EtOH to air-dry (2–3 min).

8. Repeat steps B5–7 for all mice to be tested. Once completed, return the mice to their home cage.

9. Return mice to the vivarium.

**Pause point:** The interval between familiarization (day 2) and recognition/test session (day 3) can vary from 10–15 min for short-term working memory assessment to 24 h and more for longer-term memory and time-of-day-dependent learning test, depending on the needs of the experiment.

**C. Day 3: Test session (24 h post-familiarization)**

1. Turn on the source of illumination (visible light, Figure 1A-7, or infrared light, Figure 1A-8), depending on the time of day, and ensure correct angle/brightness. Connect the camera (Figure 1A-4) to the computer and focus the lens until a clear image is obtained.

*Note: Light and camera setup must be ready* ***prior*** *to bringing the mice into the testing room.*

2. Transport mice from the vivarium to the behavioral testing room in their home cage. Obtain an additional, new (clean) cage to be used as a holding cage that will temporarily house tested mice to prevent interactions between tested and naïve mice.

3. Allow mice to acclimate to the room in their home cage for 1 h.

4. Replace one of the objects from familiarization day (day 2) with a new object [Figure 1A (2, 3); Figure 1B], placed in the same location as the familiar object. Ensure ≥ 5 cm distance between the object and the arena wall(s).

5. Begin video recording: Place the mouse (one at a time) into the arena (ensure the mouse is placed in the middle of the arena but facing away from the objects) and allow it to explore for 10 min.

6. After 10 min, stop recording and relocate the mouse from the arena to their holding cage (clean cage).

7. Wipe down the arena and objects with 70% EtOH and paper towel, allowing EtOH to air-dry (2–3 min).

8. Repeat steps C5–7 for all mice to be tested.

9. Return mice to the vivarium, collect brain tissue for further processing, or euthanize.

**Critical:** To ensure that downstream histological/biochemical analysis of mouse tissue occurs at the same time of day as behavior, collect mouse tissue 24 h after the last behavioral session.

**Videos 1–4. Examples of NOR test during light and dark phase recordings.** See also Figure 2. **Video 1** shows the light phase [zeitgeber time (ZT) 5; AM] recording of experimental day 2 (familiarization, two identical objects). **Video 2** shows the light phase recording of day 3 (recognition, novel object). The recordings are processed with DeepLabCut and BehaviorDEPOT software, showing how the exploration and kinematics data are collected. Colorful dots labeling the mouse nose, head, and tail base are used to gather tracking information (see Data analysis section). When exploration is detected, a flashing notification (banner) appears on the screen, color-coded by object number (yellow for object 1, blue for object 2, see Figure 2). **Videos 3–4:** Same as Videos 1–2, but for dark phase (ZT17; PM) recordings. All videos are cropped to show 2 min (out of the total 10 min) of recording.

**Video 1.**
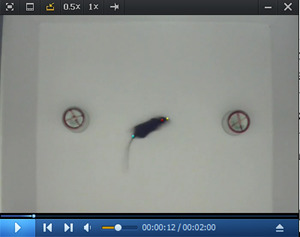
Light phase (AM)_Day2

**Video 2.**
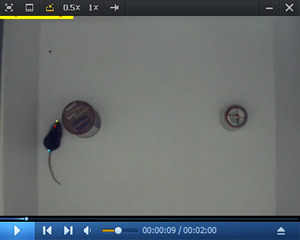
Light phase (AM)_Day3

**Video 3.**
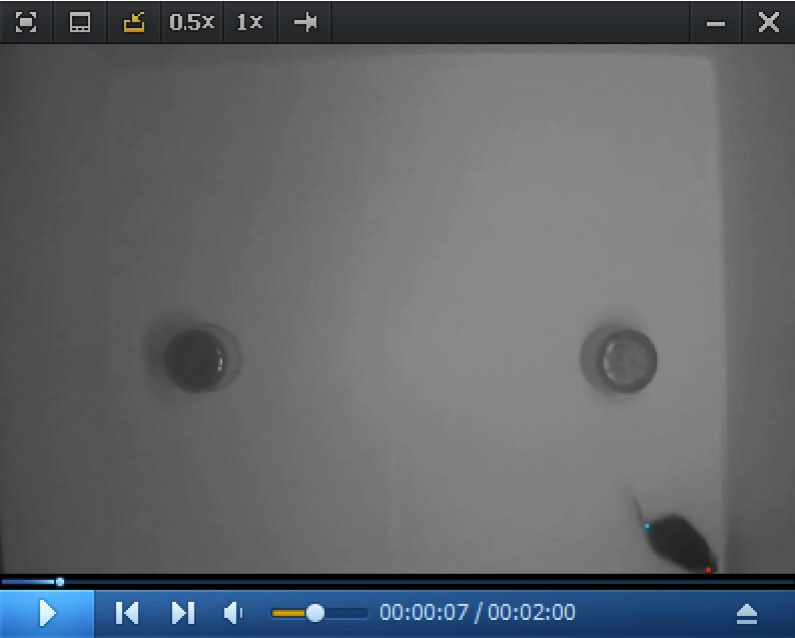
Dark phase (PM)_Day2

**Video 4.**
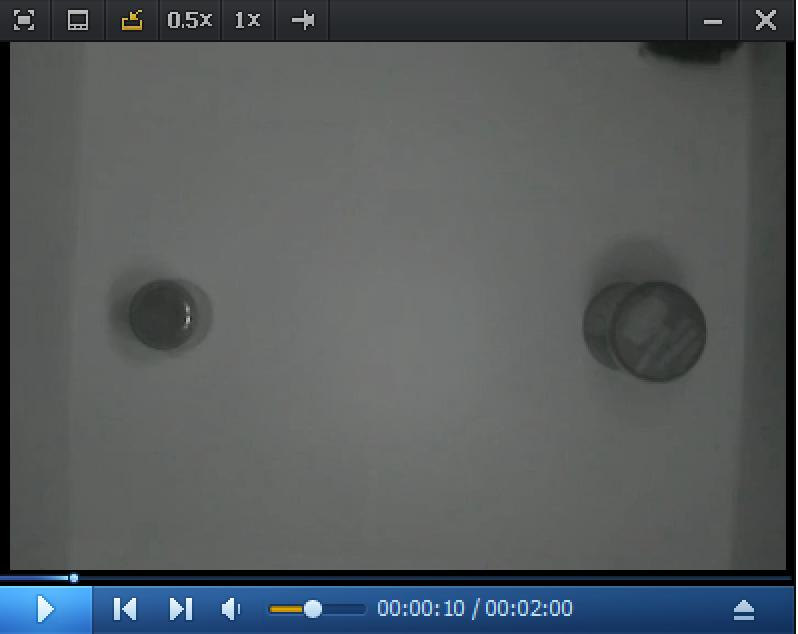
Dark phase (PM)_Day3

## Data analysis

**A. Object exploration analysis**

Mouse performance is analyzed by comparing the differential exploration of two objects. We define an exploration bout when the mouse’s head is oriented toward the object, and its nose remains within 2 cm of the object for at least 0.125 s (Videos 1–4; see “Automated scoring for object exploration” below). Exploration can be quantified in two ways: 1) calculating the total exploration time for each object, or 2) calculating the discrimination index (DI) using the following equation: DI = (T_obj1_−T_Obj2_)/(T_obj1_+T_Obj2_), where T_obj1_ and T_Obj2_ are the total time spent exploring objects 1 and 2, respectively (see Validation of protocol section). On day 3 of the experiment (Test session), object 1 is replaced with a novel object.

**A.1 Manual scoring of object exploration:** Can be performed either live during testing using a stopwatch to mark exploration bouts, or offline from recorded videos. The open-source tool Chronotate [48] can be used to obtain timestamps of exploratory behavior. For each video, the timestamps are exported as a .csv file and processed using a custom Python script for total exploration time/DI calculations (Github).

*Note: Data presented in this protocol were obtained by the automated scoring method (described below), while manual scoring was used for comparison and for calibrating parameters for automated scoring.*

**A.2 Automated scoring of object exploration**: We use a custom DeepLabCut (DLC) model [45,46,49,50] (GitHub link to DLC user guide), followed by BehaviorDEPOT [47] (Figure 3), to quantify exploration. A DLC model was trained from 7,300 images, where 9 *keypoints* (e.g., body parts such as nose, head, and tail base) were manually labeled (GitHub). The output from DLC is a .csv file containing frame-wise X-Y coordinates for each tracked body part (colored dots in Figure 2 and Videos 1–4, showing the nose, head, and tail base tracked). This file, along with its source video recording, is the input for BehaviorDEPOT, which requires several steps to be performed for analysis (Figure 3). First, the run box associated with *calculateExploration* classifier is checked (Figure 3, step 1). This classifier has three associated parameters that define exploration: minimum duration of behavior bout (Params.Exploration.minDuration; default: 0.25 s), distance of region around object to include (Params.Exploration.objDistThresh; default: 2 cm), and distance of nose within original object ROI to exclude (Params.Exploration.noseDist; default: 2 cm). In our experiments, parameters were adjusted to produce the most accurate quantification of exploration based on manual observations, and were set to: Params.Exploration.minDuration: 0.125 s, Params.Exploration.objDistThresh: 2 cm, Params.Exploration.noseDist: 0.1 cm. Note that these parameters should be modified as applicable for each experimental setup by checking the edit box associated with *calculateExploration* classifier (Figure 3, step 1, column *Edit*). Next, the Data Prep Options section is completed, which depends on the user’s specific behavioral experiment setup and DLC model used for video analysis (Figure 3, step 2). Next steps are to check boxes under *Plot Display Options* to obtain desired visualizations (Figure 3, step 3) and to slide Use GPU to ON under the *Parallel Processing* section (Figure 3, step 4). Next, both boxes are checked under the *Modules to Run* section (Figure 3, step 5), and DLC (csv) is selected under the *Tracking File Type* section (Figure 3, step 6). The appropriate option under *Start Data Analysis* section (Figure 3, step 7) is then chosen, followed by clicking *Start* (Figure 3, step 8) to begin analysis, which begins with a prompt to draw a ROI around the object and then match the specific body part labels to corresponding BehaviorDEPOT labels.

*Critical: *calculateExploration* classifier only works for one object per session, requiring each video to be analyzed twice. After exploration of both objects has been quantified, MATLAB, Python, and Excel Office Scripts/VBA macros are used to format output data and calculate the total exploration times or DIs (GitHub).

**B. Locomotion analysis (kinematics)**

X-Y coordinates obtained from DLC are smoothed in BehaviorDEPOT and used to calculate the total distance traveled (cumulative sum of distance traveled per frame) and velocity [using the equation: sqrt((dx/dt)+(dy/dt)]. In addition, BehaviorDEPOT contains a *calculateOFT* classifier that quantifies time spent in the center vs. edges of the arena to obtain information about the general state of anxiety, as typically done in the open field test [47]. This classifier can be run simultaneously with *calculateExploration* classifier to streamline analysis.

**C. Visualization**

DI/exploration/kinematic plots can be generated using graphing software of choice, such as Microsoft Excel, GraphPad Prism, or R. For quality control in addition to visualization, it is suggested to generate videos that contain both tracking data and object exploration labels overlaid upon source video by running the *auto_overlay.m* MATLAB code located within the *automated* folder of GitHub (as in Videos 1–4; Figure 2).

## Validation of protocol

To validate this protocol, two cohorts of adult male and female mice (4 months old) were subjected to the 3-day test as described in the Procedure section during the light phase [subjective night for the mice, ZT5; number of mice (N) = 8] or during the dark phase (subjective day for mice ZT17; N = 8). No sex differences were observed using this protocol; thus, male and female data were pooled. Discrimination index (DI), total exploration time for each object, and kinematics data including velocity and distance traveled were calculated using the abovementioned methods and plotted in Figure 4 and Figure S1.

For both light and dark phase experiments (termed AM and PM), we observe a significant increase in DI on day 3 compared to day 2 (data shown as median with [range]: AM day 2, -0.18 [-0.38–0.33], day 3, 0.28 [-0.33–0.45]; PM day 2, -0.04 [-0.28–0.27], day 3, 0.37 [0.03–0.61]) (Figure 4A), indicating that mice preferred exploring the novel object over the familiar object. Accordingly, no difference in total exploration time of the objects was observed on day 2 (familiarization). However, on day 3, exploration time for the familiar object (object 2) was significantly lower than for the novel object (object 1) both during the light and dark phases (data shown as median with range (exploration time in s): AM day 2, object 1, 8.25 [1–25.6], object,2, 15.45 [0.9–27.8]; day 3, object 1 (novel object), 10.75 [0.5–56.8], object 2, 6.75 [1.8–13.7]; PM day 2, object 1, 16.15 [4.8–23.9], object 2, 15.45 [4.5–22.3]; day 3, object 1 (novel object), 14.05 [7.8–27.2], object 2, 7 [1.3–18.9]) (Figure 4B). Kinematics analysis revealed no differences in locomotor activity, including travel distance (Figure 4C) and velocity (Figure 4D), between experimental days or light/dark phases (data shown as mean ± SEM: distance (cm) AM, day 2, 3858 ± 401, day 3, 3237 ± 236.8; PM, day 2, 3669 ± 328.1, day 3, 3733 ± 319.2; velocity (cm/s) AM, day 2, 6.85 ± 0.7, day 3, 5.77 ± 0.41; PM, day 2, 7.19 ± 0.56, day 3, 6.68 ± 0.54), suggesting that the exploration differences observed did not stem from deficits in locomotor activity or anxiety (which can reduce exploration by reducing overall mobility). Furthermore, exploration times or locomotion did not significantly vary across the 10-min session on either testing days or light phases (quantified by 2-min segments; Figure S1), confirming the adequacy of this session length for reliable assessment of the behavior.

## General notes and troubleshooting

**General notes**

This behavioral test assesses the cognitive function of mice, which are **extraordinarily** sensitive to stress, which can significantly impact exploratory behavior. **Critical:** All efforts to minimize stress on mice should be made.

1. Experimenter

a. Experimenters should not apply any overly fragrant cosmetics at any point during this protocol and should be comfortable with handling mice.

b. To reduce variability caused by the experimenter’s presence, the same person should ideally perform the entire experiment (days 1–3), or days 2–3 at least.

c. There should be a clear distance separating the experimenter from the arena. In the setup described here, the distance from the arena to the experimenter is 5 feet. If space permits, a sliding curtain can be installed to further isolate the mouse from the experimenter.

2. Testing room: Experiments should be conducted in a room dedicated specifically to behavioral testing. Ideally, the room is isolated from other rooms and located in a low-traffic area to minimize disturbances and noise.

3. Testing arena

a. The arena used here is constructed according to the protocol in Denninger et al. [51]. While cost-effective, it is not required, as many commercial arenas are available, for example, from Maze Engineers (https://maze.conductscience.com).

b. It is recommended that all interior surfaces of the arena have a matte finish to reduce glare and visual distractions. We accomplished this by lining the arena walls with white matte finish wallpaper (see Equipment section for details).

4. Lighting considerations

a. Strong illumination can induce anxiety in mice, reducing exploration, while disruptions in object recognition memory in mice have been reported when bright light (350 lux) was used [52]. Therefore, careful arrangement of light sources and their intensity is crucial for light phase experiments. Arrange diffuse lighting by utilizing multiple light sources (Figure 1A) to illuminate the arena for a light intensity of ~20 lux [8].

**Caution:** A diffuse lighting arrangement may result in uneven illumination across the arena. Our setup produces ~50% variance in light intensity between the front and back end of the arena; however, this has not affected mice's locomotion or exploration behavior.

b. Lighting arrangements above the arena may cause the objects to cast shadows (such as shown for IR illumination in Videos 3–4). We did not observe any changes in exploratory behavior that could be related to these shadows, such as mice avoiding or spending more time in shadowed areas.

c. If changes in behavior as a result of uneven lighting or shadows occur, adjust lighting arrangements to minimize intensity variations or shadow appearance.

5. Objects for exploration

a. Objects should be heavy/large enough to remain stationary when being explored by the mice. If needed, add a small piece of odor-free adhesive to the bottom surface of the object to stick it to the arena floor (see Equipment section item #11).

b. Use non-reflective objects that are, at a minimum, comparable in size to the mice under study. Avoid relying on color as the primary distinguishing feature between familiar and novel objects due to dim lighting conditions and the mouse’s limited visual acuity. Keep object heights comparable to prevent bias and avoid mixing climbable and non-climbable objects.

c. For these experiments, the following objects were used: 100 mL beakers (height: 6.75 cm, diameter: 5 cm), 250 mL media storage bottles (height: 10 cm, diameter: 5.9 cm), and an empty cylindrical food tin (thoroughly sanitized and cleaned with 70% EtOH; height: 12 cm, diameter: 6.5 cm). The beakers and bottles are made non-reflective by covering exposed surfaces with masking tape. See Figure 1B and [53] for further guidance regarding object properties.

*Note: While only cylindrical objects are used in this protocol, rectangular and/or square-shaped objects can also be used, as long as they maintain the characteristics described above.*

d. Objects should be placed on opposite sides of the arena with a minimum of 5 cm between the object and the arena wall. Caution should be taken if objects are to be placed in the corners of the arena, as mice prefer to groom themselves in these areas. Grooming behavior occurring in an object-containing corner may be misidentified as exploration when using automated scoring.

**Caution:** Mice should not face either object when placed into the arena.

6. Video recordings

a. For optimal recordings, the camera should be mounted directly above the arena with the lens perpendicular to the arena floor (Figure 1A-1, 4; distance from camera to the arena floor is 96.5 cm). Since an overhead view is essential, a camera with a varifocal lens is highly recommended for fine-tuning the field of view. When mounted, the camera should be at a height where, when recording, the arena occupies the entire field of view.

b. The optimal focal length varies depending on the source of illumination. When switching illumination sources (such as for IR vs. visible light), the lens will need to be readjusted.

c. Due to adjustments required when switching from visible to IR illumination, the use of an adhesive to secure the camera to the stand is not recommended.

d. For light phase recordings, multiple small lamps (e.g., under-cabinet LED lights; see lighting considerations above) that face away from the arena are optimal (Figure 1A-7).

e. For dark phase recordings, a webcam with a low-light-capable image sensor must be used. Avoid cameras with built-in IR filters. IR illuminators should be installed above and facing downward onto the arena floor (Figure 1A-8; see lighting considerations above).

7. Computational considerations/data analysis software

a. Ensure your computer has a sufficient GPU to train a neural network in DeepLabCut (DLC).

b. It is highly recommended that a customized DLC model be generated that fits the specific conditions of the experiment, such as mouse size, light conditions, and camera setup. Use the DeepLabCut Model Zoo platform to fine-tune the model using your labeled data to improve a custom model’s performance. Furthermore, for best model performance, it is advised to crop novel videos before DLC analysis to contain only the testing arena within the field of view, as outside objects may interfere with DLC tracking.

c. BehaviorDEPOT can use tracking data generated by SLEAP [54] in lieu of DLC. Both programs have the same hardware requirements; thus, the decision on which software to use is experimenter-dependent.

d. As an alternative to DLC-based analysis, we have utilized an open-source behavioral classification pipeline, “Circadian Behavioral Analysis Suite” (CBAS) [55], to classify exploratory behaviors with a high degree of confidence. This method is advantageous as it allows for combined tracking and behavioral classification within the same toolkit, streamlining the analysis process.

8. Data interpretation and subjects’ behavior variability

a. Mice show high variability in exploration time and, sometimes, bias toward one object or area of the arena, which can skew the results and impact data interpretation. To ensure a high degree of reproducibility and reduce variability, at least 8–10 mice per experimental group should be used.

b. Consider using set criteria for excluding mice from the testing group, such as total exploration time or DI. For example, if total exploration time is below X seconds (the exact number should be determined experimentally), this can indicate reduced locomotion, while DI above X number during familiarization day can indicate bias to an object/side of the arena. *Note: We did not apply any such exclusion criteria for data presented in this protocol (Figure 4, Figure S1) to demonstrate the “natural” variability.*

c. If mice exhibit significant differences in locomotion and/or exploration during the 10-min session, consider analyzing the data in small time segments (as shown in Figure S1) and removing segments that show significantly different activity to reduce variability.

d. To reduce bias for an object or arena side, consider shifting the position of the novel object between tested individuals.

e. To further reduce variability, strive for consistency of the experimental conditions such as time of day, sex (test male and female mice in separate cohorts, preferably on different days), noise levels, experimenter, and mice origin (avoid mixing mice raised under different conditions, such as laboratory vivarium and a commercial source).

**Troubleshooting**

**Problem 1**: Mice spend more time traveling along the perimeter of the arena and are not exploring objects/mice are not moving around in the arena.

Possible cause 1: The testing environment is anxiogenic.

Solution 1.1: Monitor the conditions of the behavioral experiment room while the mouse is in the arena for noise. If there are brief, intermittent bursts of noise (people conversing while walking past the room, noise from pipes and/or vents), a white noise machine may be able to mask these disturbances. If there are chronic, audible disturbances, it would be best to relocate to a quieter area.

Solution 1.2: Ensure that illumination is below 350 lux and check that lights are not directed toward the arena (see Figure 1A) and that the lighting arrangements do not generate strong illumination gradients (more than 2-fold difference in lux) across the arena or the objects cast large shadows.

If the issue persists and the source of the anxiogenic stimulus cannot be identified, consider placing the arena inside a sound attenuation chamber, as reported in Cordeira et al. [56].

Possible cause 2: Decreased exploration, which we observed to be characteristic of older mice (≥4 months old).

Solution 2: Use larger objects when assessing older adult mice (as in Figure 1B). Alternatively, reduce the time interval between familiarization (day 2) and test day (day 3) from 24 h to 15 min to 1 h. **Caution:** This modification will assess working memory instead of intermediate/long-term memory.

Possible cause 3: Mouse genetic background. Certain inbred mouse strains have been shown to exhibit strain-dependent differences in both exploratory and anxiety-like behaviors [57,58].

Solution 3: If applicable, switch the mouse background strain to increase exploratory behavior. Alternatively, try handling the mice (5–10 min of contact in the vivarium) 2–3 days prior to the experiment to reduce the stress of human presence.

**Problem 2**: Mice spend more time exploring one object compared to the other during familiarization day (day 2) or spend more time on one side of the arena than the other.

Possible cause: Bias to object location within the arena or to the object itself.

Solution: Test for object bias by allowing mice to explore different sets of object pairs. Discard any object for which multiple mice consistently spend more time exploring. If mice exhibit a side or location bias, consider shifting the position of the novel object between individual mice, or try rotating the object positions by 90°, placing objects that were along the longer horizontal walls along the shorter vertical walls. If the bias persists, rotate the entire arena by 90°.

**Problem 3**: Poor visibility at night.

Possible cause: Insufficient number of IR illuminators used.

Solution: Initial night-time experiments were recorded using a single IR illuminator and produced poor-quality videos that were unsuitable for automated scoring. To properly illuminate the entire arena, multiple IR illuminators are required (Figure 1A-8).

## Supplementary information

The following supporting information can be downloaded here:

1. Figure S1. Validation of the NOR protocol.
